# Colorimetric Point-of-Care Detection of *Clostridium tyrobutyricum* Spores in Milk Samples

**DOI:** 10.3390/bios11090293

**Published:** 2021-08-24

**Authors:** Paola Cecere, Francesca Gatto, Claudia Cortimiglia, Daniela Bassi, Franco Lucchini, Pier Sandro Cocconcelli, Pier Paolo Pompa

**Affiliations:** 1 Nanobiointeractions & Nanodiagnostics, Istituto Italiano di Tecnologia (IIT), 16163 Genova, Italy; paola.cecere@iit.it (P.C.); francesca.gatto@iit.it (F.G.); 2 Dipartimento di Scienze e Tecnologie Alimentari per la Sostenibilità della Filiera Agro-Alimentare, Facoltà di Scienze Agrarie Alimentari ed Ambientali, Università Cattolica del Sacro Cuore, Via Emilia Parmense 84, 29122 Piacenza-Cremona, Italy; claudia.cortimiglia@unicatt.it (C.C.); daniela.bassi@unicatt.it (D.B.); franco.lucchini@unicatt.it (F.L.); pier.cocconcelli@unicatt.it (P.S.C.)

**Keywords:** *Clostridium tyrobutyricum*, spores, LAMP, point-of-care, colorimetric test, milk

## Abstract

*Clostridium tyrobutyricum* represents the main spoiling agent responsible for late blowing defects (LBD) in hard and semi-hard cheeses. Its spores are resistant to manufacturing procedures and can germinate during the long ripening process, causing the burst of the cheese paste with a consequent undesirable taste. The lower quality of blown cheeses leads to considerable financial losses for the producers. The early identification of spore contaminations in raw milk samples thus assumes a pivotal role in industrial quality control. Herein, we developed a point of care (POC) testing method for the sensitive detection of *C. tyrobutyricum* in milk samples, combining fast DNA extraction (with no purification steps) with a robust colorimetric loop-mediated isothermal amplification (LAMP) technique. Our approach allows for the sensitive and specific detection of *C. tyrobutyricum* spores (limit of detection, LoD: ~2 spores/mL), with the advantage of a clear naked-eye visualization of the results and a potential semi-quantitative discrimination of the contamination level. In addition, we demonstrated the feasibility of this strategy using a portable battery-operated device that allowed both DNA extraction and amplification steps, proving its potential for on-site quality control applications without the requirement of sophisticated instrumentation and trained personnel.

## 1. Introduction

The contamination of milk by endospores represents a critical issue for industrial quality control in the production of hard and semi-hard cheese, such as Grana Padano, Parmigiano Reggiano, Emmental, and Gouda. Although milk does not provide suitable growth conditions, long ripening times characteristic of these cheeses may create favorable anaerobic conditions for the germination of spores in vegetative cells [1]. The resulting metabolic activity leads to pronounced cheese spoilage defects. Late blowing is one of the most frequent problems still affecting hard cheese production in the dairy industry. Such spoilage is characterized by butyric fermentation of lactate achieved by the vegetative cells, leading to the production of butyric acid, acetic acid, hydrogen, and carbon dioxide [2,3]. As a consequence, late blowing results in the burst of the cheese load, generation of several heterogeneously distributed cavities (corresponding to the volumes of gas produced and mass digested), and a resulting undesirable taste. The lower sensory quality of blown cheeses leads to significant financial losses for producers [4]. Among spore-forming anaerobic bacteria, butyric acid-producing clostridia are considered the main agents of such damages, due to their spore-surviving attitude during whole-cheese manufacturing including heat treatments [5,6,7,8,9,10,11]. Several clostridial species, mainly *Clostridium tyrobutyricum*, *Clostridium butyricum*, *Clostridium sporogenes*, and *Clostridium beijerinckii*, have been isolated from blown cheese [9,10,12,13,14]. In particular, *C. tyrobutyricum* has been identified as the principal spoiling organism responsible for late blowing defects (LBD) [7,9,15,16,17,18,19,20,21]. The avoidance of such issue is challenging, due to the ubiquitous presence of *Clostridium* spores. The main sources of contamination are thought to be silage, water, and unhygienic animal bedding [22,23]. Moreover, although LBD occurs mostly in unpasteurized milk, even pasteurized milk cheeses may be affected.

Several approaches have been proposed to prevent clostridia spoilage, including the use of additives [24], milk bactofugation or microfiltration [25,26], or the addition of lactic acid bacterial strains biologically active against Gram-positive bacteria [27,28,29,30,31]. However, most of these methods have technical or regulatory limitations. Traditionally, cow’s milk is processed through consolidated manufacturing practices, which lead to the production of typical products registered as a Protected Designation of Origin cheeses (PDO, European Union). Preservatives like nitrate and lysozyme may be added to milk during cheese production, due to their capacity to selectively reduce the microorganism survival and multiplication [16,24,32,33]. Nitrate was commonly employed in Emmental production [34], but its use was banned after the decision of the European Food Safety Authority (EFSA) to reduce levels of nitrosamines in food products (EFSA, 2010). In Italy, lysozyme from egg is the only bacteriostatic agent allowed for the protection of raw cow’s milk in Grana Padano cheese production. Its addition seemed to influence bacterial distribution in terms of *Clostridium* and *Lactobacillus*. In particular, *C. tyrobutyricum* was negatively affected by the presence of lysozyme [35].

The bacterial communities associated with the spoiled hard cheese have been studied through routine diagnostic methods, involving microbiological methods (most probable number (MPN) procedures), molecular approaches (PCR-based methods and Next-generation sequencing (NGS) technology), and immunological methods (immune-assays, flow cytometry) [9,14,16,18,36,37]. The identification of specific bacterial species using conventional cultivation methods is a long and difficult process, involving MPN counting and subsequent confirmation by checking the lactate fermentation capacity of cells from gas-positive samples [6,38]. This method is arduous for adapting to the needs of the production process, due to the requirement of several days for the identification of positive samples. Additionally, it is not possible to discriminate between the different species using specific media or phenotypic markers [39,40], and a series of further tests is frequently necessary.

To overcome these issues, different strategies for detecting species-specific DNA sequences of spoiling bacteria in milk have been devised. Currently, standard methods include DNA extraction from spores and its subsequent amplification using PCR or real-time PCR-based techniques [7,35,36,41,42]. However, the extraction of spore DNA from food matrices poses several limitations, starting from the necessity for long and multi-step procedures. Indeed, to achieve an efficient lysis of spores, it is often necessary to combine the use of chemical buffers with the mechanical disruption of spores through bead beating or sonication, which requires extra instrumentation and specialized laboratories. In addition, an important issue is the common occurrence of false-negative results due to the presence of various food substances and chemicals used for the extraction that can inhibit the PCR reaction [30]. Furthermore, PCR-based techniques are still too costly for routine applications in the cheese industry, due to the need of specialized instrumentation and skills [4]. In this framework, the development of a rapid, effective, and low-cost method for on-site diagnostics would be of great importance. 

In this work, we have developed a point-of-care (POC) molecular method to track contaminations of *C. tyrobutyricum* spores in milk samples. In particular, our test exploits a simple and direct DNA extraction step through the fast chemical lysis of spores in heating conditions, without the need for mechanical disruption and/or purification procedures, coupled to the spore detection through a colorimetric loop-mediated isothermal amplification (LAMP). The identification of contaminated samples was proven to be possible by a simple visual inspection, while achieving a highly sensitive, specific, and semi-quantitative detection. The feasibility of the entire process for on-site applications was demonstrated using a portable battery-operated heating device, suitable for both the lysis and the amplification steps.

## 2. Materials and Methods

### 2.1. Clostridium tyrobutyricum Spore Preparation 

*C. tyrobutyricum* UC7086 from Università Cattolica Culture Collection (Piacenza, Cremona, Italy) was used for the development of the LAMP assay and for the milk artificial contamination to evaluate the performance of the method. *C. tyrobutyricum* spores were obtained using the protocol described by Bassi et al. [21]. Briefly, the strain was cultured in reinforced clostridial medium broth (RCM) (Oxoid, Altrincham, UK) and incubated at 37 °C in anaerobiosis. 1% of well-grown suspension was inoculated into a regenerated cellulose tubular membrane located in a bottle with 400 mL of RCM broth. After five days of anaerobiosis at 37 °C and at least 15 days of aerobiosis, the membrane content was checked to verify the presence of a high percentage of spores by visualization with phase-contrast microscopy (Nikon, Tokio, Japan). Spores were then collected, washed several times with sterile distilled water, and purified using Percoll gradient (Merck KGaA, Darmstadt, Germany) as previously described [43]. No vegetative cells were observed at the phase-contrast microscopy. Purified spores were stocked at −20 °C. The spore counts were determined by plating 10-fold dilutions of the obtained spore solution after pasteurization for 10 min at 80 °C. The stock was used to develop the LAMP assay and to artificially inoculate milk samples. 

### 2.2. Artificial Contamination and Milk Samples Preparation

Raw milk was collected and stored at 20 °C before use. The volume was divided into aliquots of 20 mL, which were inoculated with 1000, 100, and 10 spores. A negative control was also added consisting of milk without spores. Each sample was treated with 20 mL of Buffer F (EDTA 100 mM (Sigma-Aldrich, St. Louis, MO, USA), sodium citrate 250 mM (Carlo Erba, Cornaredo, Italy), Tris HCl 200 mM (Sigma-Aldrich, St. Louis, MO, USA), Triton X-100 0.01%; final pH 7.5 (Sigma-Aldrich, St. Louis, MO, USA)) and incubated in a water bath at 65 °C for 1 h. After that, samples were vortexed for 10 s and centrifuged at 13,000 rpm for 30 min at 40 °C. The supernatant was discarded, paying attention to avoid the mixing of pellets with the residual fat. The experiment was performed in triplicates. Moreover, an aliquot of each sample was analyzed to check the right number of recovered spores, through the plating of decimal dilutions on RCM agar (Oxoid, Altrincham, UK).

### 2.3. Specificity and Sensitivity of LAMP Assay

To determine the sensitivity of the assay, serial dilutions of the aqueous spore stock were prepared and tested by real-time fluorescent detection. To determine the specificity of the reaction, several relevant strains were used. In particular, two additional strains of *C. tyrobutyricum* (UC9037, UC9038) and different clostridial, namely *C. sporogenes* (UC9000) and *C. butyricum* (*UC9034* and *ATCC10702*), and nonclostridial species commonly present in milk (*Lactobacillus acidophilus UC8810*, *Lactobacillus rhamnosus UC8887*, *Lactobacillus reuteri UC8916*, *Lactobacillus sakei UC8577*, *Leuconostoc lactis UC8773*, *Lactococcus lactis MG1363*, *Lactobacillus plantarum UC8491*, *Escherichia coli UC4131*) were selected for this purpose. *Clostridium* species were inoculated in RCM broth (Oxoid, Altrincham, UK) in anaerobiosis at 37 °C, *Lactobacillus* and *Leuconostoc* were cultivated in MRS broth (Oxoid, Altrincham, UK) in anaerobiosis at 37 °C, *E. coli* was cultivated in LB broth (Oxoid, Altrincham, UK) in aerobiosis at 37 °C. The genomic DNA of these strains was extracted from pure culture using the EZNA bacterial DNA kit (Omega Bio-tek, GA, USA) following the manufacturer’s instructions. 

### 2.4. Rapid DNA Extraction 

The obtained pellets were resuspended in 100 µL of lysis buffer (InstaGene^TM^ Matrix, Bio-Rad, München, Germany) and briefly vortexed. The samples were then heated at 130 °C for 7 min in a prototypal battery-operated device. After 7 min incubation time, the samples were left to cool down for a few minutes and were directly processed in the LAMP reactions, with no purification steps.

### 2.5. Primer Design

LAMP primers were designed from the phosphotransacetylase (*pta*) gene of *Clostridium tyrobutyricum*, and the univocal alignment was verified using the Basic Local Alignment Search Tool (BLAST). Primer sequences are reported in Table 1. 

### 2.6. LAMP Reactions for Real-Time Fluorescence Detection

LAMP reactions were performed in a 25 µL final volume using the protocol described by Cibecchini et al. [44] with the following modifications: 1.6 µM of forward inner primer (FIP) and backward inner primer (BIP), 0.2 µM of forward outer primer (F3) and backward outer primer (B3), 0.4 µM of forward loop primer (LF) and backward loop primer (LB) (Integrated DNA Technologies, Coralville, IA, USA), and 10 µL of DNA template. DNA-free LAMP reactions were included as negative controls. Real-time amplifications were conducted at 63 °C on the StepOne^TM^ Applied Biosystem Real-Time PCR instrument (Thermo Fisher Scientific, Waltham, MA, USA) using the StepOne^TM^ Software v2.3. 

### 2.7. LAMP Reactions for Colorimetric Assay

LAMP colorimetric reactions were performed in a 50 µL final volume following the protocol described by Cibecchini et al. [44] with some modifications: 1.6 µM each of inner primers, 0.2 µM each of outer primers, 0.4 µM of loop primers (Integrated DNA Technologies, Coralville, IA, USA), and 20 µL of DNA template. DNA-free LAMP reactions were included as negative controls. Colorimetric LAMP reactions were performed on a prototypal portable battery-operated device at 63 °C for 1 h, and the amplification efficiency was verified by the pink-to-yellow color change of the reaction mix.

## 3. Results and Discussion

**Figure 1:**
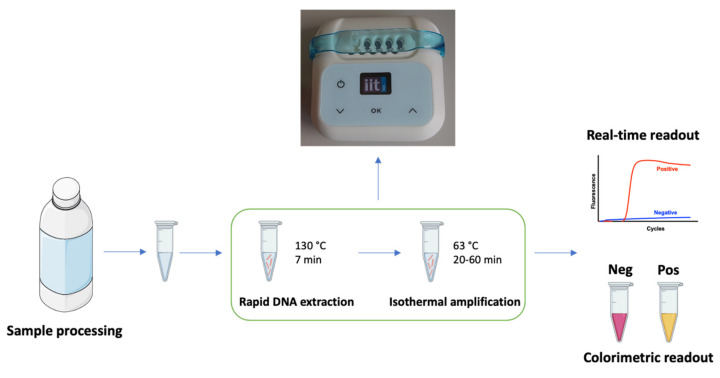
Schematic workflow of the POC process for *C. tyrobutyricum* identification. The method comprises three steps: milk sample processing, rapid DNA extraction performed at 130 °C for 7 min, and DNA isothermal amplification performed at 63 °C for 20–60 min, with a fluorescent or naked-eye colorimetric readout.

The milk spoilage caused by *C. tyrobutyricum* represents a crucial issue in the field of cheese production, affecting long-ripened hard cheese losses. To address this concern, the present study focused on the design and development of a rapid POC method aimed at detecting the presence of *C. tyrobutyricum* spores in milk samples. The schematic workflow of the entire procedure is displayed in Figure 1.

Raw milk samples were artificially contaminated with *C. tyrobutyricum* spores obtained from pure culture. 20 mL spiked samples were centrifuged to obtain spore pellets. A rapid DNA extraction procedure was developed and optimized using a simple lysis buffer and a “super-heating” step (above 100 °C), thus avoiding the complex mechanical lysis of the spores. The heating treatment was carried out by systematically investigating different operating temperatures, between 100 and 160 °C with 5 °C increment steps, and different incubation times, in order to achieve optimal lysis conditions. Interestingly, when using 130 °C heating conditions, we managed to achieve an efficient DNA extraction from the spore pellets in only 7 min, a very convenient method for on-site quality control applications. Lower temperatures produced an incomplete lysis, whereas higher temperatures led to DNA degradation. The lysis procedure was also effectively performed using a basic prototypal battery-operated heating device.

The resulting solution was then directly used for the amplification phase, without any purification steps due to the robustness of the LAMP reaction and the absence of any inhibitor in the lysis buffer. An aliquot of the extracted DNA from the super-heating-treated tubes was directly added to the amplification mix. 

**Figure 2:**
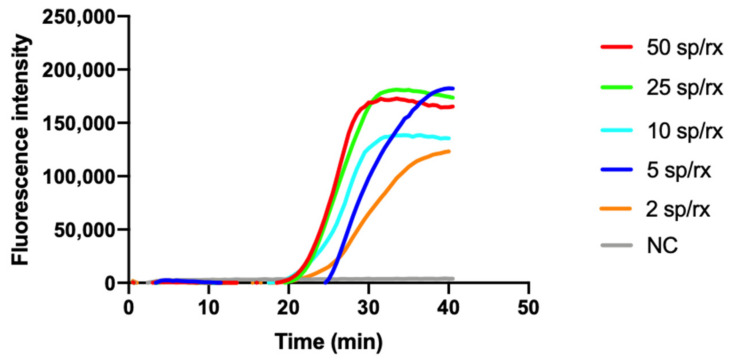
Representative SYBR Green-based real-time fluorescence detection of *C. tyrobutyricum* spores in water at decreasing concentrations of spores/reaction (sp/rx). NC: negative control. All experiments were performed in quadruplicate, each being repeated at least three times.

To assess and optimize the efficiency of the lysis step and the sensitivity of the isothermal reaction, we performed preliminary experiments extracting *C. tyrobutyricum* spores dispersed in water. The real-time fluorimetric LAMP reaction showed a high sensitivity, demonstrating that the developed heating procedure guaranteed the efficient lysis of the samples. The method was proven to achieve an excellent limit of detection (LoD) as low as 2 spores/reaction and a very fast readout (ca. 20 min), as reported in Figure 2.

**Figure 3:**
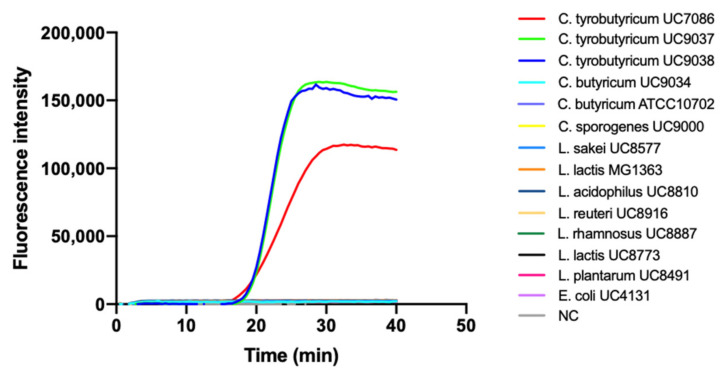
SYBR Green-based real-time fluorescent analysis of LAMP specificity based on genus-specific primer for *C. tyrobutyricum*. NC: negative control.

We then verified the specificity of the LAMP assay by testing the reaction with genomic DNA from different clostridia species (*C. sporogenes* and *C. butyricum*) and other nonclostridial species usually contaminating raw milk, namely *Lactobacillus acidophilus*, *Lactobacillus rhamnosus*, *Lactobacillus reuteri*, *Lactobacillus sakei*, *Leuconostoc lactis*, *Lactococcus lactis*, *Lactobacillus sakei*, *Lactobacillus plantarum*, and *Escherichia coli.* Interestingly, our technique was found to be highly specific, recognizing only the *C. tyrobutyricum* species, with no false positive results detected (Figure 3).

After the optimization of our test, we attempted to develop a simplified and instrument-free approach with a naked-eye readout through a clear color change of the reaction samples. To this end, we exploited the pH-sensitive LAMP strategy using the Cresol Red dye, which results in a pink-to-yellow color change upon target amplification, enabling the identification of positive samples by simple visual inspection [44,45,46,47]. 

**Figure 4:**
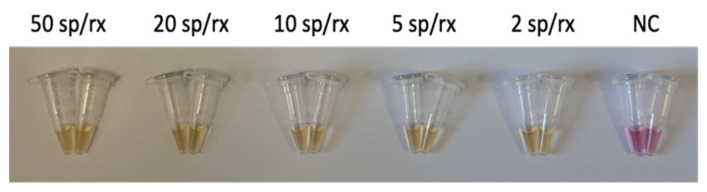
Representative colorimetric LAMP results for *C. tyrobutyricum* spore detection in water at different spore/reaction (sp/rx) concentrations. NC: negative control. All experiments were performed in quadruplicate, each being repeated at least three times.

After colorimetric LAMP, the reaction preserved the same sensibility displayed in the real-time fluorimetric detection, with a LoD of 2 spores/reaction. A representative result is shown in Figure 4. The colorimetric assay was able to identify the *C. tyrobutyricum* contamination, exhibiting a clear pink-to-yellow visible color change. Notably, the amplification reaction was effectively performed in the portable battery-operated device developed in our laboratories, revealing an opportunity for carrying out the entire method in point-of-care settings without the need for sophisticated instrumentations.

**Figure 5:**
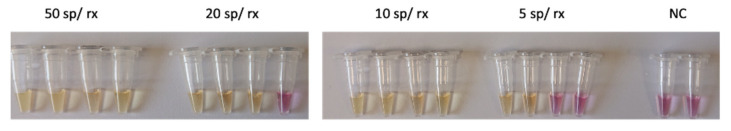
Representative colorimetric LAMP results for *C. tyrobutyricum* spore detection in milk samples. NC: negative control. All experiments were performed in quadruplicate, each being repeated at least three times.

Once it was assessed that the colorimetric procedure was able to consistently detect the presence of *C. tyrobutyricum* spores even at a low concentration, we investigated its implementation in milk samples using artificially contaminated specimens and the same extraction procedure described above with no purification steps. In these tests, we took advantage of the high tolerance of the LAMP reaction to matrix contaminants [45]. As displayed in Figure 5, the experimental results showed the achievement of a LoD of 10 spores/reaction, with a 50% detection reliability for 5 spores/reaction. This indicates a minor loss of sensitivity in the case of real milk samples using the fast POC methodology.

Taking a further step, we explored the possibility of developing a semi-quantitative variant of our colorimetric test in order to address the industrial need for the quantification of *C. tyrobutyricum* levels in milk samples, which has practical relevance in the production process of hard cheese.

**Figure 6:**
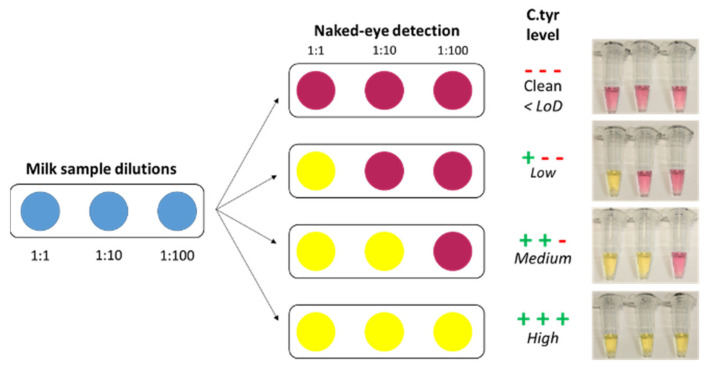
Semi-quantitative colorimetric test. Sample serial dilution allows for the identification of four different levels of contaminations. Zero yellow tubes: no contamination; one yellow tube: low contamination (ca. 10–100 spores); two yellow tubes: medium contamination (ca. 100–1000 spores); three yellow tubes: high contamination (ca. >1000 spores). A representative photo of a real experiment with 0, 20, 200, 2000 sp/rx (from top to bottom) is shown on the right.

As explained in Figure 6, we exploited a visual detection scheme, using serial dilutions (1:1, 1:10, 1:100) of the milk samples, and we tested them through the colorimetric technique, obtaining three tubes relative to the different dilutions of each sample. Depending on the number of yellow tubes (positive tests), we can infer the level of spore contamination in a semi-quantitative way, i.e., a low (one positive tube), medium (two positive tubes), or high (three positive tubes) level of contamination, while noncontaminated samples have zero yellow tubes. Based on the sensitivity of our colorimetric method (10 sp/rx LoD), this strategy can roughly discriminate the <10, 10–100, 100–1000, or >1000 spore contamination ranges. A test experiment with milk samples spoiled with 0, 20, 200, and 2000 spores/reaction confirmed the reliability of the devised strategy (Figure 6, right).

## 4. Conclusions

In this work, we reported the design and development of a fast POC strategy that combined a simple thermochemical DNA extraction, based on a fast purification-free “super-heating” step, with a colorimetric LAMP technique for the rapid naked-eye detection of *C. tyrobutyricum* spores in milk samples. The LAMP amplification, an efficient isothermal reaction operated in a simple and portable heating device, demonstrated enough sensitivity and specificity for quality control purposes. The direct colorimetric visual readout makes this approach potentially useful for on-field applications in the industry and hard cheese production chain.

## Figures and Tables

**Table 1:** Names and sequences of primers used in this study.

Name	Sequence (5′–3′)
F3	GAGAACTTCAATTGGATGCTTC
B3	TTGCAGCTTGTGCTTGTA
FIP	AGGTCCTATTGCTTCCGCTTGCACCTGGTAGTCCTGTA
BIP	TGTCAAGGCTTTGCAAAACCAACTGCTGTTACAGCTACTACATT
LoopF	TTCCTGCTTGAAGTTCAGG
LoopB	GAGGATGCAGTTCTGACGATAT

## Data Availability

The data presented in this study are available on request from the corresponding author.
